# Investigating the Diagnostic Utility of LncRNA GAS5 in NAFLD Patients

**DOI:** 10.3390/biomedicines13081873

**Published:** 2025-08-01

**Authors:** Maysa A. Mobasher, Alaa Muqbil Alsirhani, Sahar Abdulrahman Alkhodair, Amir Abd-elhameed, Shereen A. Baioumy, Marwa M. Esawy, Marwa A. Shabana

**Affiliations:** 1 Department of Pathology, Biochemistry Division, College of Medicine, Jouf University, Sakaka 72388, Saudi Arabia; 2 Department of Chemistry, College of Science, Jouf University, Sakaka 72341, Aljouf, Saudi Arabia; amassaf@ju.edu.sa; 3 Department of Biochemistry, Faculty of Science, King Abdulaziz University, Jeddah 21589, Saudi Arabia; salkhodair@kau.edu.sa; 4 Internal Medicine Department, Faculty of Human Medicine, Zagazig University, Zagazig 31527, Egypt; ameer_barakat2019@outlook.com; 5 Microbiology and Immunology Department, Faculty of Human Medicine, Zagazig University, Zagazig 31527, Egypt; drshereenatef@yahoo.com; 6 Clinical Pathology Department, Faculty of Human Medicine, Zagazig University, Zagazig 31527, Egypt; dr.marwaesawy@ymail.com (M.M.E.); marwa_shabana@yahoo.com (M.A.S.)

**Keywords:** biomarker, lncRNA GAS5, microRNA-29a-3p, NOTCH2, NAFLD patient

## Abstract

**Background/Objectives**: Non-alcoholic fatty liver disease (NAFLD) is one of the most common chronic liver conditions globally. This study aimed to assess the long non-coding RNAs (lncRNAs) growth arrest-specific 5 (GAS5), miR-29a-3p, and neurogenic locus notch homolog protein 2 (NOTCH2) as biomarkers in patients with NAFLD and find out if they are related to any clinical factors. **Subjects and Methods**: Thirty-eight age-matched healthy persons and thirty-eight NAFLD patients were enrolled. Patients were split into the following three groups: non-alcoholic steatohepatitis (NASH) (n = 12), patients with NAFLD-related cirrhosis (n = 8), and patients with NAFLD-related simple steatosis (n = 18). Real-time PCR was utilized to examine the expression. **Results**: The lncRNA GAS5 and NOTCH2 were higher in NAFLD cases in comparison to controls. On the other hand, microRNA-29a-3p was underexpressed in NAFLD cases in comparison to controls. Regarding NAFLD diagnosis, lncRNA GAS5 was the best single marker with a sensitivity of 100% and a specificity of 94.7% at the cutoff values of ≥1.16-fold change. Regarding different stages of the disease, the highest level of lncRNA GAS5 was in cirrhosis. lncRNA GAS5 expression, among other studied parameters, is still a significant predictor of NAFLD (adjusted odds ratio of 162, C.I. = 5.7–4629) (*p* = 0.003). LncRNA GAS5 has a positive correlation with NOTCH2 and a negative correlation with miR-29a-3p. LncRNA GAS5, NOTCH2, and RNA-29a-3p were significantly different in NAFLD cases compared to controls. **Conclusions**: lncRNA GAS5 appears to be the most effective single marker for detecting NAFLD. LncRNA GAS5 expression is a significant independent predictor of NAFLD. LncRNA GAS5 can differentiate different NAFLD stages.

## 1. Introduction

Non-alcoholic fatty liver disease (NAFLD) is one of the most common chronic liver conditions globally and is frequently linked to type 2 diabetes, obesity, and unhealthy eating habits [1]. In Western countries, approximately one-quarter of the population is affected by NAFLD [2]. Triglyceride elevation in the liver that occurs irregularly is the first sign of NAFLD, which can worsen into serious liver illnesses [3]. NAFLD frequently develops slowly, starting with basic steatosis and developing over the years without noticeable symptoms. Non-alcoholic steatohepatitis (NASH), a more severe type of disease that involves inflammation and liver damage, can develop if this condition is not treated. NASH can occasionally develop into cirrhosis, fibrosis, and, eventually, hepatocellular cancer, which is one of the illnesses that make up NAFLD [4]. Moreover, it encompasses simple steatosis, which has a less harmful course [5]. It is anticipated that between 2016 and 2030, the prevalence of NASH will rise by almost 56% in many nations around the world [6].

Currently, imaging methods (including ultrasound and magnetic resonance imaging [MRI]) and invasive liver biopsy (which is associated with potential complications) are the primary tools for diagnosing and evaluating NAFLD. Several serum biomarkers are regularly examined, including lipid profiles, inflammatory markers, liver enzymes (Alanine aminotransferase (ALT) and aspartate aminotransferase (AST)), and markers related to insulin resistance and fibrosis. These markers are not sensitive or specific enough to correctly stage NAFLD or differentiate between basic steatosis and NASH. Therefore, reliable, noninvasive biomarkers that can predict the occurrence, severity, and course of disease are urgently required. Because of their stability in circulation and disease-specific expression patterns, molecular biomarkers like non-coding RNAs have been the focus of recent research.

Growing data points to the importance of long non-coding RNAs (lncRNAs) as regulators of several physiological and pathological processes, including liver functions [7]. In NAFLD there is aberrant expression of lncRNAs, which regulates the disease’s progression. In the liver tissues of mice with induced NAFLD, lncRNA GAS5 is elevated and linked to the development of hepatocellular carcinoma. Nevertheless, it is currently unknown if GAS5 controls the pathophysiology of NAFLD [8].

Moreover, steatosis is a result of the modulation of lipid accumulation within hepatocytes by certain lncRNAs, such as MEG3 and HULC, which regulate genes related to fatty acid production, oxidation, and storage. Others, like NEAT1 and MALAT1, are linked to the promotion of oxidative stress and inflammatory responses, which worsen hepatocyte damage and speed up the progression from simple steatosis to NASH. Furthermore, it has been demonstrated that lncRNAs, such as HOTAIR and APTR, stimulate hepatic stellate cells and encourage the deposition of extracellular matrix, contributing to liver fibrosis, a defining feature of the disease’s progression toward cirrhosis. Later, a number of lncRNAs, such as HULC and MALAT1, are connected to the development of hepatocellular carcinoma (HCC) because of their functions in metastasis, apoptosis resistance, and cellular proliferation [9].

Additionally, research has shown that microRNAs (miRNAs) contribute to the onset of NAFLD [10]. Potential NAFLD indicators include plasma miR-29a, which contributes to NAFLD regulation. For instance, in NAFLD, miR-29a-3p regulates lipid levels and cholesterol metabolism [11]. Transforming growth factor-β1 (TGF-β1) signaling is suppressed by NOTCH2, and this signaling is linked to the upkeep of chronic inflammation, such as NAFLD [12]. Furthermore, it has been revealed that one of miR-29a-3p’s targets is neurogenic locus notch homolog protein 2 (NOTCH2). NOTCH2 expression is known to promote the advancement of NAFLD [13].

A novel GAS5-driven process contributing to the emergence of NAFLD has been demonstrated by focusing on the miR-29a-3p/NOTCH2 pathway. The selection of growth arrest-specific 5 (GAS5), miR-29a-3p, and NOTCH2 as biomarkers for NAFLD can be attributed to their involvement in key biological processes such as inflammation, fibrosis, and cellular metabolism, all of which are central to the pathogenesis of NAFLD. Additionally, their expression levels can reflect disease progression, making them potential targets for diagnostic and therapeutic strategies. Research continues to explore their specific roles and mechanisms to better understand their utility in clinical settings [14].

Even though non-invasive imaging technologies like ultrasonography are frequently employed currently to diagnose NAFLD, reliable molecular biomarkers are still needed to improve early detection and support current approaches. Finding molecular markers may also provide information on the underlying disease pathways, which could help direct future targeted therapy approaches. So, this study aimed to assess lncRNA GAS5, miR-29a-3p, and NOTCH2 expression as biomarkers for NAFLD and determine the existence of their clinical associations.

## 2. Materials and Methods

### 2.1. Study Design and Subjects

Patients from Zagazig University Hospitals were the subjects of case–control research. The Institutional Review Board (IRB) of the Faculty of Human Medicine at Zagazig University (IRB number #11156), which was established in accordance with the Declaration of Helsinki, verified the study. The sample size was calculated using statistical powers and confidence intervals (CIs). Assuming the expression levels of lncRNA GAS5 in cases are 1.3 ± 0.55 and are 1 ± 0.35 in controls, the calculated sample size is 76 subjects. In the current study, 38 age-matched healthy individuals and 38 patients with NAFLD were enrolled between November 2023 and August 2024. NAFLD cases were chosen using an expert’s evaluation of ultrasonographic data in accordance with the American Gastroenterology Association’s established standards [15]. The exclusion criteria included alcohol intake, Wilson’s disease, autoimmune hepatitis, and viral hepatitis.

Additionally, patients were omitted if took oral contraceptives, methotrexate, tamoxifen, or corticosteroids. Patients with thyroid diseases, pregnancy, and cancer were not included. Although the initial symptoms ranged widely, the most prevalent ones were nausea and vomiting (26.3%). Fatigue, skin itching, and upper abdomen pain were other complaints.

Once written informed consent was received, all included patients had liver biopsies to confirm their inclusion. Subsequently, patients with NAFLD were categorized into the following three groups: non-alcoholic steatohepatitis (NASH) with 12 participants, cirrhosis with 8 participants, and simple steatosis with 18 participants.

### 2.2. Methods

#### 2.2.1. Routine Laboratory Tests

Routine laboratory tests were conducted using a Cobas 8000 device (c702 module) from Roche Diagnostics in Mannheim, Germany. These tests included a fasting lipid profile, measuring total cholesterol (TC), triglycerides (TG), low-density lipoprotein cholesterol (LDL-C), and high-density lipoprotein cholesterol (HDL-C), as well as liver enzymes, specifically alanine aminotransferase (ALT) and aspartate aminotransferase (AST).

#### 2.2.2. RNA Extraction

A serum/Plasma Kit (QIAGEN, GmbH, Hilden, Germany) was applied following the manufacturer’s guidelines. The amount and purity of the extracted RNA were determined by a NanoDrop 2000 spectrophotometer (Thermo Scientific, Waltham, MA, USA).

#### 2.2.3. cDNA Synthesis

In a reverse transcription experiment, the RNA template is first converted into complementary DNA (cDNA) using the enzyme reverse transcriptase (RT), which requires one microgram of RNA and is performed using a miScript RT II kit in accordance with QIAGEN GmbH (Hilden, Germany) manufacturer’s instructions.

#### 2.2.4. Quantitative Real-Time PCR

Real-time PCR was utilized to examine the expression on a step one real-time PCR machine, made in Foster City, CA, USA, by Applied Biosystems, by utilizing a miScript SYBR Green PCR kit. The thermal protocol for RT-qPCR was outlined as follows: the first denaturation stage was followed by a total of 40 amplification cycles starting with heating at 95 °C for 15 s, then at 58 °C for 30 s, followed by 70 °C for 30 s. The specificity of the reaction was verified by an annealing curve analysis. GAPDH and U6 were utilized as reference genes to normalize the expression. We calculated the relative expression of miR and lncRNA using the 2^−ΔΔCT^ calculation method. Matching controls with patients during relative gene expression analysis typically involves selecting controls that are similar in key variables such as age and sex to reduce confounding. The primers used are shown in Table 1. The primer sequences are based on previously published protocols and are designed to specifically amplify targets [14].

### 2.3. Statistical Analysis

The analysis of the data was conducted using SPSS version 20 (Chicago, IL, USA). For quantitative data, an independent sample *t*-test along with a one-way ANOVA with a post hoc Tukey test were conducted to analyze the data. The Chi-square test is applied appropriately for qualitative data. An investigation into the relationship between the marker and other factors was performed using a Pearson correlation analysis. The chosen markers’ diagnostic efficacy was evaluated using the receiver operating characteristic (ROC) curve, which was calculated after the disease status had been assessed. Univariate followed by multivariate analysis were performed to identify the predicted variables. Values less than 0.05 showed statistical significance.

## 3. Results

### 3.1. Characterization of Subjects

We assessed the demographic and laboratory data of each subject recruited for the current study, including those in the case group (n = 38) and the control group (n = 38). Table 2 indicates that there was significant dyslipidemia in NAFLD patients when compared to the healthy, age-matched control group. Table 2 reveals that the examined groups differed significantly in terms of BMI, ALT, and AST. Our results revealed significantly higher expression levels of lncRNA GAS5 and NOTCH2 in NAFLD cases compared to controls.

### 3.2. Marker Levels

**Figure 1:**
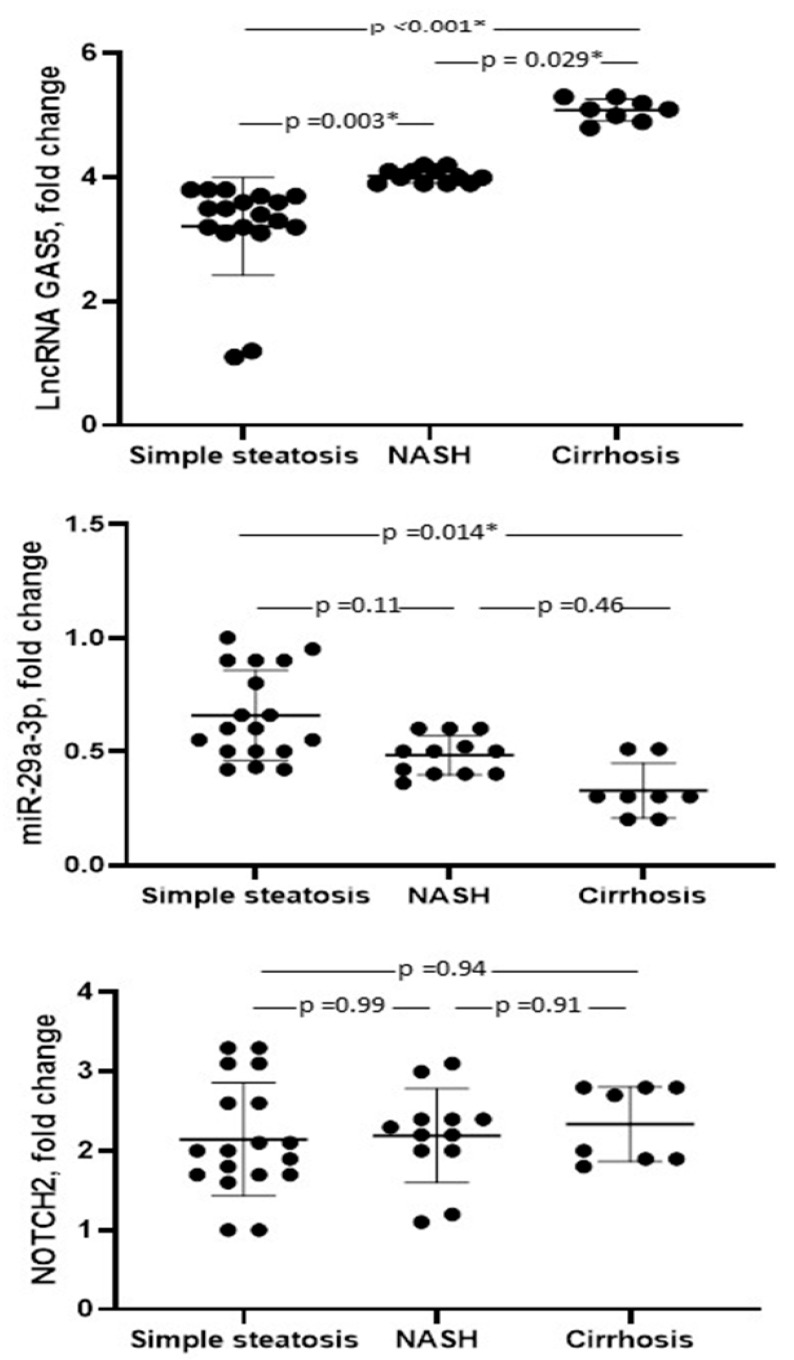
Boxplots present the levels of biomarkers in different types of NAFLD [steatosis, non-alcoholic steatohepatitis (NASH), and cirrhosis]. GAS5: growth arrest-specific 5, miR-29a-3p: microRNA-29a-3p, NOTCH2: notch receptor 2. *: Significant.

On the other hand, microRNA-29a-3p was underexpressed in NAFLD cases in comparison to controls. Regarding the different stages of the disease, the highest level of lncRNA GAS5 was observed in cirrhosis (5.13 ± 0.17), NASH (4.1 ± 0.1), and simple steatosis (3.18 ± 0.62) (*p* < 0.001, Figure 1). However, the actual differences in lncRNA GAS5 expression (e.g., from 3.18 to 5.13) may appear modest. To prove that the detected differences between groups are sufficiently robust for reliable clinical application, the effect size was calculated. The effect size [Pearson’s r] was 0.72 and 0.96 in differentiating steatosis from NASH and in differentiating NASH from cirrhosis, respectively. These results indicated a large effect size [Pearson’s r > 0.50], which means high reliability and potential clinical utility for lncRNA GAS5 in monitoring disease progression. The power of sample size among subgroups was calculated by the normal approximation method. Regarding GAS5, in a comparison between different patients’ subcategories using a two-sided 95% confidence interval the power is indicated as 100%. The calculated power suggested a high likelihood of detecting a true difference if it exists and is accepted for medical research (>80%).

Regarding microRNA-29a-3p expression levels, there is a significant difference between simple steatosis and cirrhosis (0.67 ± 0.23 and 0.33 ± 0.13, respectively). NOTCH2 showed no significant difference in different stages of the disease (*p* = 0.91).

### 3.3. Diagnostic Performance of the Marker

**Figure 2:**
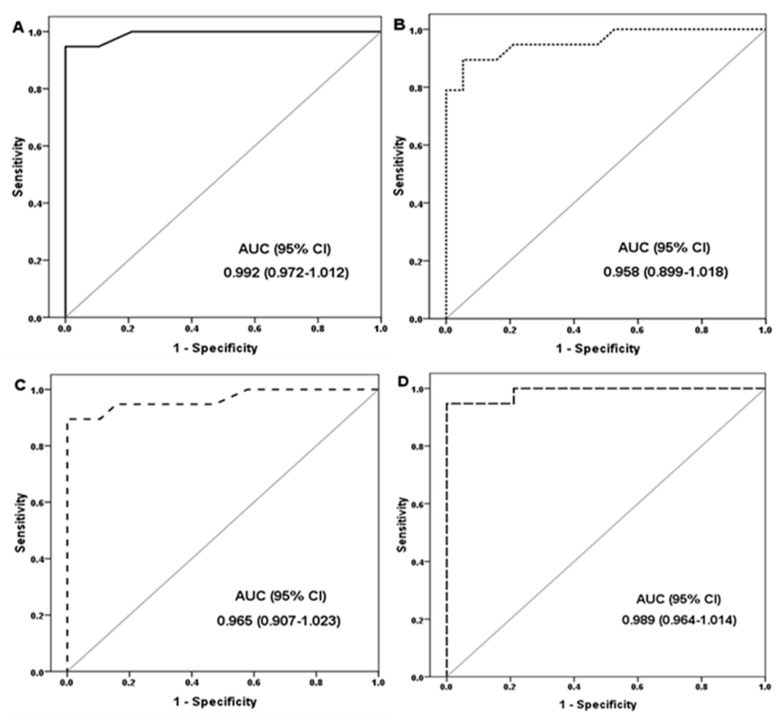
Receiver operating characteristic (ROC) curve analysis for the prediction of NAFLD using various biomarkers. (**A**) lncRNA GAS5 expression levels, (**B**) microRNA-29a-3p levels, (**C**) NOTCH2 expression levels, (**D**) Combination of lncRNA GAS5, microRNA-29a-3p, and NOTCH2. AUC: Area under curve; CI: confidence interval.

We analyzed our findings to assess the diagnostic roles of the markers studied by ROC curves and assessed their AUCs, which are illustrated in Figure 2. The markers’ diagnostic performance criteria are presented in Table 3. LncRNA GAS5 was the best single marker, with a sensitivity of 100% and a specificity of 94.7% at the cutoff values of ≥1.16-fold change. Moreover, GAS5 had statistically significant differences across disease stages (*p* < 0.001). ROC curve analysis of GAS5 revealed a 3.75-fold change, achieving 100% sensitivity and 83.3% specificity in differentiating steatosis from NASH. Additionally, a 4.5-fold change showed 100% sensitivity and 100% specificity in differentiating NASH from cirrhosis. These findings suggest that GAS5 has strong potential as a reliable biomarker for differentiating various stages of NAFLD.

### 3.4. Correlation Analysis of the Studied Markers

There were positive relationships found between the expression levels of lncRNA GAS5 and NOTCH2 and variables such as BMI, total cholesterol, LDL-C, and liver enzyme levels (Table 4). miR-29a-3p showed significance with the same parameters but with a negative correlation. LncRNA GAS5 is positively correlated with NOTCH2 and negatively correlated with miR-29a-3p.

### 3.5. Logistic Regression Analysis of MVC Risk Factors

We further investigated our findings using a logistic regression test. We include key correlators (BMI, total cholesterol, and LDL-C) with the studied marker (Table 5). Interestingly, all parameters were predictors of NAFLD in the univariate analysis. In the multivariate model, only lncRNA GAS5 expression, among other studied parameters, is still a significant predictor of NAFLD, with an adjusted odds ratio of 162 (95% CI: 5.7–4629; *p* = 0.003). This suggests that GAS5 may serve as a more specific and potentially early biomarker for identifying individuals at risk of NAFLD before clinical diagnosis, offering advantages over traditional markers like LDL or BMI alone.

## 4. Discussion

Excessive hepatic triglyceride accumulation is a hallmark of NAFLD. A variety of disease mechanisms define NAFLD. Hepatocellular carcinoma (HCC) is one possible end-stage liver disease that arises from the condition eventually. NAFLD patients suffer from serious health and financial issues [16].

The role of dyslipidemia in those with NAFLD was examined because these patients had significantly greater levels of dyslipidemia than the healthy control group. Furthermore, notable lipoprotein anomalies in NALFD were discovered by Amor et al.’s 2019 NMR spectroscopy study [17]. In atherogenic dyslipidemia, which is characterized by plasma hypertriglyceridemia, Deprince et al. [18] also demonstrated a decrease in HDL-C levels and an increase in tiny, dense LDL particles. Previous studies have shown that most NAFLD patients also have metabolic syndrome (MS). Consequently, NAFLD is thought to be the hepatic manifestation of MS. The relationship between NAFLD and lipid MS is becoming more and more clear, and the etiology of NAFLD is known to involve obesity, type 2 diabetes mellitus (T2DM), and dyslipidemia [19].

Real-time PCR was utilized to examine the expression of markers studied by the relative analysis method, which is more straightforward and widely applicable, especially when the primary goal is to compare expression levels between groups. This is in contrast to absolute quantification that requires generating standard curves with known copy numbers, which can be technically demanding and time-consuming.

There were statistically significant variations in BMI, ALT, and AST between the groups under analysis. Similar results were validated by Huang et al. [20], who found that high levels of AST and ALT are non-invasive markers of NAFLD. Li et al. [21] found that individuals with NAFLD had significantly higher average or median age, BMI, ALT, AST, LDL-C, and non-HDL-C in comparison to those without the disease. NAFLD occurred more frequently in men than in women. The risk of NAFLD was found to be significantly positively correlated with the atherogenic index [21].

Advanced hepatic fibrosis is the key prognostic indicator for NAFLD. Patients with cirrhosis have reduced levels of GAS5, a long non-coding RNA (lncRNA) associated with the inhibition of hepatic fibrogenesis. Patients with advanced fibrosis had GAS5 expression levels higher than healthy people. As fibrosis became more advanced, so did the level of GAS5 expression in plasma. Before cirrhosis develops, elevated levels of the circulating lncRNA GAS5 are linked to liver fibrosis [22]. Our results showed that lncRNA GAS5 and NOTCH2 were significantly higher expressed in NAFLD cases than in controls. Moreover, there has been evidence from a previous study suggesting that GAS5 downregulation could be a useful therapeutic approach for the management of NAFLD. GAS5 overexpression resulted in an increase in hepatic lipid accumulation. In both in vitro and in vivo models of fatty liver, GAS5 was activated [23].

Research has demonstrated that miR-29a can decrease liver damage in specific situations, such as NAFLD. The effect of miR-29a is hepatoprotective. Furthermore, we found that, compared to controls, microRNA-29a-3p was underexpressed in NAFLD patients, and mounting data suggest that NAFLD/NASH development may be prevented in part by miR-29a [24].

The three disease phases with the highest levels of lncRNA GAS5 were cirrhosis, NASH, and simple steatosis. The expression levels of microRNA-29a-3p are significantly different in simple steatosis and cirrhosis. NOTCH2 showed little variation throughout the disease’s different stages.

A growing body of evidence suggests that overexpressing GAS5 in vivo, which sponges miR-29a-3p, increases liver cell NOTCH2 levels and accelerates the development of NAFLD. Additionally, by inhibiting NOTCH2, miR-29a-3p prevented the progression of NAFLD in vivo. All things considered, these results revealed a novel GAS5-mediated mechanism behind the onset of NAFLD and indicated that GAS5 might be a viable target for NAFLD treatment. GAS5 functions as a sponge for miR-29a-3p, increasing NOTCH2 expression and advancing the development of NAFLD [14].

Serum miR-29 levels and liver fibrotic stages are negatively correlated according to earlier studies on how miR-29 contributes to liver fibrosis [25]. Furthermore, a later study revealed that GAS5 may contribute to the transformation of NAFLD into HCC [26]. Whether or not the patient had extensive fibrosis determines which traits belong to which category. Severe fibrosis patients showed higher AST levels, reduced platelet counts, and a greater risk of developing diabetes compared to people without the illness. Moreover, they were far older.

Regarding GAS5 expression analyses, plasma GAS5 levels in individuals with advanced fibrosis were notably greater than those in patients without the condition. The presence or severity of NASH did not significantly affect GAS5 expression. The degree of fibrosis was strongly linked with tissue GAS5 expression. Moreover, Han et al. [22] discovered a stronger positive correlation between plasma levels and the fibrosis stage.

We examined our results to determine the ROC curves’ diagnostic roles for the examined indicators as well as their AUCs. The diagnostic performance criteria of the markers were evaluated. The most effective single marker was lncRNA GAS5, which had a 100% sensitivity and 94.7% specificity at cutoff values of >1.16-fold change.

There were significant positive correlations between lncRNA GAS5 and NOTCH2 expression levels and BMI, as well as total cholesterol, LDL-C, and liver enzymes. miR-29a-3p showed significance with the same parameters but with a negative correlation. LncRNA GAS5 is positively correlated with NOTCH2 and negatively correlated with miR-29a-3p. Utilizing a logistic regression test, we investigated our findings further. We include key correlators (BMI, total cholesterol, and LDL-C) with the studied marker. Interestingly, all parameters were predictors of NAFLD in the univariate analysis. In the multivariate model, only lncRNA GAS5 expression, among other parameters studied, is still a significant predictor of NAFLD. GAS5 may be a biomarker for NAFLD and provide protection against the development of NAFLD [27].

In fatty liver models, GAS5 was activated both in vitro and in vivo. Hepatic lipid accumulation was exacerbated by GAS5 overexpression, but lipid droplet accumulation was reduced by GAS5 knockdown. Research has shown that hepatic lipid accumulation can be decreased by downregulating GAS5, indicating that this may be a promising therapeutic strategy for how NAFLD is managed [23].

Regarding the different stages of the disease, the highest level of lncRNA GAS5 was observed in cirrhosis, followed by NASH, and then simple steatosis. Studies have demonstrated that lncRNA GAS5 may play a vital regulatory role in the occurrence and development of organ fibrosis, including liver, renal, and cardiac fibrosis. One study by Han et al. [22] showed that both liver and plasma lncRNA GAS5 expression were positively correlated with the progression of liver fibrosis in 51 patients with NAFLD, and this was more apparent in plasma. Also, they found that as liver fibrosis progressed, plasma and tissue GAS5 levels increased. In the animal model, lncRNA GAS5 depletion reduced lipid accumulation in the mice and reversed the elevated NAFLD activity score. Moreover, lncRNA GAS5 knockdown attenuated high-fat diet (HFD)-enhanced serum triglycerides and total cholesterol levels and relieved HFD-caused elevation of serum AST and ALT levels. These results demonstrate that LncRNA GAS5 promotes NAFLD progression in vivo. LncRNA GAS5 knockdown attenuated HFD-induced hepatic steatosis, and this attenuation was reversed by the miR-29a-3p inhibitor; thereby providing vital evidence to establish the function of lncRNA GAS5 in the pathogenesis of NAFLD and its progression by targeting miR-29a-3p in vivo [14].

Limitations must be taken into consideration, including the study’s rather modest sample size. Thus, it is advised to conduct more research with larger sample sizes in the future. The therapeutic response was not assessed, which requires further studies. Gene expression at the RNA level may not always directly correlate with protein levels, especially for lncRNAs, which often exert their functions through interactions rather than encoding proteins. While our current study focuses on the lncRNA GAS5 expression profile, another limitation in the study was the inability to incorporate proteomic analyses and functional assays, which could provide a more comprehensive understanding of its roles and enhance the robustness of its application in clinical settings.

## 5. Conclusions

LncRNA GAS5, NOTCH2, and RNA-29a-3p were significantly different in NAFLD clinical cases compared to controls. LncRNA GAS5 seems to be the best single marker in NAFLD detection. LncRNA GAS5 expression is a significant independent predictor of NAFLD. LncRNA GAS5 can differentiate different NAFLD stages.

## Figures and Tables

**Table 1:** Sequences of the forward and reverse primers that were utilized in this study.

Target	Forward Primer	Reverse Primer	Tm
GAS5	5′-GTGTCTCTCTCTCTCTCTCTCTT-3′	5′-CCTCTTCAGCAGTAGCATAGTT-3′	57.9
NOTCH2	5′-GTGGCATACTGGGAGGAGAA -3′	5′-GATGGAGAAACCAGGGAACA-3′	57.9
miR-29a-3p	5′-GCACCGTCAAGGCTGAGAAC-3′	5′-CAGCCCATCGACTGGTG-3′	59
GAPDH	5′-ACCAGGAAATGAGCTTGACA-3′	5′-GACCACAGTCCATGCCATC-3′	57.6
U6	5′-ACACGCACAAACGAGAAAGG-3′	5′-AGTGCAGGGTCCGAGGTATT-3′	59.5

Tm: Melting temperature, GAS5: growth arrest specific 5, NOTCH2: notch receptor 2, miR-29a-3p: microRNA-29a-3p, GAPDH: glyceraldehyde 3-phosphate dehydrogenase, U6: U6 small nuclear RNA.

**Table 2:** The characteristics of subjects.

Parameters	NAFLD (No.: 38)	Controls (No.: 38)	*p*
Age, years	45 ± 6.4	45.4 ± 6.8	0.83
Sex (Female/Male)	30/8 (78.9/21.1)	28/10 (73.7/26.3)	0.70
BMI, Kg/m^2^	29.3 ± 2.6	25.8 ± 2.1	<0.001 *
Total cholesterol, mg/dL	169.3 ± 53.9	130.2 ± 35	0.012 *
Triglyceride, mg/dL	98.7 ± 24.5	92.2 ± 20.5	0.44
HDL-C, mg/dL	46.8 ± 6.1	43.7 ± 5.4	0.10
LDL-C, mg/dL	103.1 ± 52.6	69.7 ± 31.8	0.023 *
AST (IU/L)	52.7 ± 16.4	23.4 ±5.6	<0.001 *
ALT (IU/L)	69.5 ± 17	23.9 ± 7.5	<0.001 *
LncRNA GAS5, fold change	3.9 ± 0.95	1.04 ± 0.07	<0.001 *
miR-29a-3p, fold change	0.53 ± 0.21	0.99 ± 0.09	<0.001 *
NOTCH2, fold change	2.2 ± 0.63	1.03 ± 0.13	<0.001 *

NAFLD: Non-alcoholic fatty liver disease; BMI: body mass index; HDL-C: high-density lipoprotein cholesterol; LDL-C: low-density lipoprotein cholesterol; AST: aspartate aminotransferase; ALT: alanine aminotransferase; GAS: growth arrest-specific; NOTCH2: neurogenic locus notch homolog protein 2. Data was expressed as mean ± SD or frequency (parentage). *: Significant.

**Table 3:** Markers’ diagnostic performance criteria.

Marker	Cutoff (Fold Change)	Youden’s Index	Sensitivity (%)	Specificity (%)	PPV (%)	NPV (%)	Accuracy (%)
LncRNA GAS5	≥1.16	0.95	100	94.7	95	100	97.4
miR-29a-3p	≤0.91	0.89	89.4	94.7	94.4	90	92.1
NOTCH2	≥1.4	0.84	89.4	100	100	90.5	94.7
Combined lncRNA GAS5, microRNA-29a-3p, and NOTCH2	Same cutoffs	0.95	94.7	100	100	95	97.4

PPV: Positive predictive value; NPV: negative predictive value.

**Table 4:** Correlation analysis of the studied markers.

Parameters	LncRNA GAS5		miR-29a-3p		NOTCH2	
	r	*p*	r	*p*	r	*p*
Age	−0.02	0.89	−0.01	0.94	−0.03	0.86
BMI	0.62	<0.001 *	−0.55	<0.001 *	0.42	0.009 *
Total cholesterol	0.39	0.014 *	−0.17	0.28	0.31	0.06
Triglyceride	0.17	0.29	−0.05	0.75	0.15	0.37
HDL-C	0.16	0.34	−0.06	0.74	−0.04	0.98
LDL-C	0.35	0.031 *	−0.14	0.41	0.28	0.09
AST	0.78	<0.001 *	−0.69	<0.001 *	0.67	<0.001 *
ALT	0.82	<0.001 *	−0.71	<0.001 *	0.68	<0.001 *
miR-29a-3p	−0.91	<0.001 *	1	-	−0.78	<0.001 *
NOTCH2	0.8	<0.001 *	−0.78	<0.001 *	1	-

r: Correlation coefficient; BMI: body mass index; HDL-C: high-density lipoprotein cholesterol; LDL-C: low-density lipoprotein cholesterol; AST: aspartate aminotransferase; ALT: alanine aminotransferase; NOTCH2: neurogenic locus notch homolog protein 2. *: Significant.

**Table 5:** Logistic regression analysis of risk factors.

Variables	Univariate		Multivariate	
	OR (95% CI)	*p*	AOR (95% CI)	*p*
BMI	1.8 (1.2–2.6)	0.001 *	1.93 (0.91–4.1)	0.09
Total cholesterol	1.02 (1–1.04)	0.026 *	1.02 (0.78–1.32)	0.90
LDL-C	1.02 (1–1.04)	0.039 *	0.99 (0.73–1.35)	0.95
LncRNA GAS5	324 (18.7–5588)	<0.001 *	162 (5.7–4629)	0.003 *
miR-29a-3p	0.01 (0.001–011)	<0.001 *	0.1 (0–8.3)	0.25
NOTCH2	72 (9–573)	<0.001 *	1.9 (0.02–159)	0.76

OR: Odds ratio; AOR: adjusted odds ratio; CI: confidence interval; BMI: body mass index; LDL-C: low-density lipoprotein cholesterol; NOTCH2: neurogenic locus notch homolog protein 2. *: Significant.

## Data Availability

Data will be available on request to Dr. Marwa A. Shabana, Clinical Pathology Department, Faculty of Human Medicine, Zagazig University, Egypt.

## Abbreviations

The following abbreviations are used in this manuscript:

NAFLD	Non-alcoholic fatty liver disease
NASH	Non-alcoholic steatohepatitis
HCC	Hepatocellular carcinoma
lncRNAs	Long non-coding RNAs
TC	Total cholesterol
TG	Triglycerides
LDL-C	Low-density lipoprotein cholesterol
HDL-C	High-density lipoprotein cholesterol
ALT	Alanine aminotransferase
AST	Aspartate aminotransferase
cDNA	Complementary DNA
BMI	Body mass index
GAS	Growth arrest-specific
NOTCH2	Neurogenic locus notch homolog protein 2.
miRNAs	microRNAs
